# Correction: Adhesive functionalized ascorbic acid on CoFe_2_O_4_: a core–shell nanomagnetic heterostructure for the synthesis of aldoximes and amines

**DOI:** 10.1039/d1ra90171k

**Published:** 2021-11-29

**Authors:** Serve Sorkhabi, Mohammad Ghadermazi, Roya Mozafari

**Affiliations:** Department of Chemistry, University of Kurdistan P.O. Box 66135-416 Sanandaj Iran mghadermazi@yahoo.com +98 873324133 +98 87 33624133

## Abstract

Correction for ‘Adhesive functionalized ascorbic acid on CoFe_2_O_4_: a core–shell nanomagnetic heterostructure for the synthesis of aldoximes and amines’ by Serve Sorkhabi *et al.*, *RSC Adv.*, 2020, **10**, 41336–41352, DOI: 10.1039/D0RA08244A.

The authors regret that the image used for Fig. 1e in the original manuscript was from an incorrect sample.

The correct image is given here.
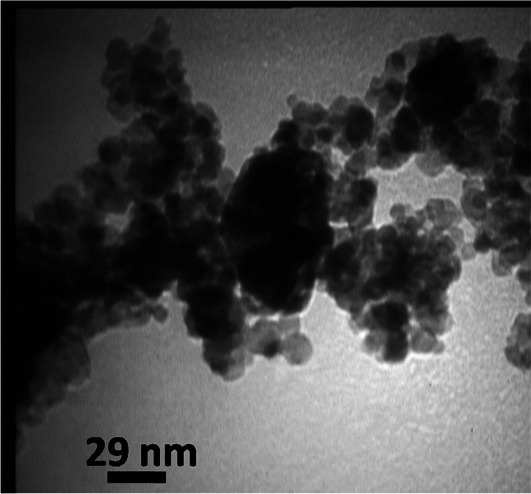


The authors state that the findings and overall conclusions presented in the original article are unaffected by this change.

The Royal Society of Chemistry apologises for these errors and any consequent inconvenience to authors and readers.

## Supplementary Material

